# Relationships Between Consumption of High-Saturated-Fat Foods, Sleep Duration, BMI, Depression, Age and Sex, and Emotional Eating in Peruvian Adolescents: A Cross-Sectional Study

**DOI:** 10.3390/nu17162662

**Published:** 2025-08-18

**Authors:** Yaquelin E. Calizaya-Milla, Ingrid Puente De La Vega-Fernández, David Javier-Aliaga, Mery Rodríguez-Vásquez, Christian Casas-Gálvez, Ana Valle-Chafloque, Jacksaint Saintila

**Affiliations:** 1 School of Human Nutrition, Faculty of Health Sciences, Universidad Peruana Unión, Lima 15472, Peru; puentedelavegafernandezingridc@gmail.com (I.P.D.L.V.-F.); davidjavieraliaga@gmail.com (D.J.-A.); meryrv@upeu.edu.pe (M.R.-V.); 2 Research Group for Nutrition and Healthy Behaviors, Universidad Señor de Sipán, Chiclayo 14000, Peru; cgalvezchristic@uss.edu.pe (C.C.-G.); vchafloqueanali@uss.edu.pe (A.V.-C.)

**Keywords:** emotional eating, adolescents, depression, saturated fat, body mass index, sleep duration, Peru

## Abstract

**Background:** Emotional eating, defined as the tendency to eat in response to emotions, has been associated with various biopsychosocial factors. However, in the Peruvian context, there is limited evidence regarding the specific predictors of this eating behavior in adolescents. **Objective:** To examine the associations between saturated fat intake, sleep duration, body mass index (BMI), depressive symptoms, age and sex, and emotional eating in Peruvian adolescents. **Methods:** This was a predictive cross-sectional study based on non-probabilistic sampling. A total of 722 adolescents from four schools located in East Lima were included. A multiple linear regression model was employed to examine the relationships between age, sex, saturated fat intake (SFI), sleep duration, BMI, and depressive symptoms and emotional eating. **Results:** The model was statistically significant (adjusted R^2^ = 0.301; F = 45.276; *p* < 0.001), explaining 30.1% of the variance in emotional eating based on the explanatory variables. Being female (β = 0.208; *p* = 0.011), aged 15 to 18 versus 12 to 14 years (β = 0.083; *p* < 0.001), having a high SFI (β = 0.186; *p* < 0.001), sleeping ≥ 7 h (β = −0.126; *p* < 0.001), and a higher BMI (β = −0.082; *p* = 0.011) were significantly associated with emotional eating. Depressive symptoms (β = 0.365; *p* < 0.001) emerged as the strongest predictor in the model. **Conclusions:** Emotional eating among Peruvian adolescents is associated with psychological, behavioral, and sociodemographic factors. Depressive symptoms showed the strongest association, while longer sleep duration was linked to lower emotional eating scores. These findings highlight the need for integrated interventions targeting mental health, sleep hygiene, and healthy dietary behaviors in adolescents.

## 1. Introduction

Adolescence is a critical period for the development of eating habits and health-related behaviors [1]. According to recent estimates, mental health problems and disordered eating patterns affect approximately 15% and 22% of adolescents worldwide, respectively, representing a growing public health concern [2,3]. In the Peruvian context, 29.6% of adolescents aged 12 to 17 are at risk of experiencing a mental or emotional health problem [4]. Only 11.3% of the Peruvian population aged 15 and older meets the fruit and vegetable intake recommended by the World Health Organization (WHO) [5]. Studies conducted among Peruvian adolescents report a prevalence of eating disorder symptoms ranging from 7.3% to 13.9%, particularly among females and those with low family meal frequency, poor family communication, or sociocultural pressures related to thinness and body image [6,7]. Recent studies indicate that 62.1% of individuals engage in emotional eating [8]. In terms of body mass index (BMI), it is estimated that 62.7% of individuals aged 15 and older are overweight [9]. This situation is exacerbated by factors such as food insecurity, lack of nutritional education, and limited access to mental health services. For example, in Peru, eight out of ten individuals do not receive mental health care despite needing it [10]. Therefore, there is a clear need to investigate the contributing factors behind these behaviors to design more effective preventive and educational interventions.

Excessive consumption of foods high in saturated fat, along with factors such as BMI, sleep quality and duration, and depressive symptoms, significantly influences the development of emotional eating. However, the literature exploring these interactions in Peruvian adolescents remains limited, despite the fact that this population faces socioeconomic and cultural conditions that may heighten their vulnerability to such maladaptive eating behaviors [4,11].

Among the many psychosocial constructs related to disordered eating, body image dissatisfaction and poor body appreciation have emerged as critical mediators between internalized social ideals and maladaptive eating behaviors [12]. These constructs, particularly relevant during adolescence, interact with emotional regulation capacities and perceived stress to shape responses such as emotional eating [13,14,15]. Gross’s Emotion Regulation Model posits that negative emotions elicit psychological distress, prompting individuals to adopt coping strategies to mitigate it—such as emotional eating [16]. From this perspective, emotional eating functions as a maladaptive regulatory mechanism that, while offering temporary relief, ultimately reinforces the cycle of emotional discomfort and dysfunctional behaviors [1]. Previous studies support this model; for instance, research conducted among Chinese adolescents found that higher levels of emotional suppression—and, in females, lower levels of cognitive reappraisal—were associated with greater consumption of energy-dense foods, with emotional eating serving as a key mediating factor [17]. Shriver et al. [1] demonstrated that poor emotional regulation in childhood predicts higher levels of emotional eating during adolescence, particularly among normal-weight adolescents and those with a negative body image. Therefore, emotional eating is a multifactorial phenomenon shaped by both emotional regulation strategies and body weight, underscoring the importance of implementing comprehensive interventions from early stages of development.

The consumption of foods high in saturated fat is a key dietary pattern linked to adverse physical and mental health outcomes [18]. This type of food, commonly found in ultra-processed products such as fried foods, cold cuts, industrial baked goods, and fast food, is characterized by high energy density and low nutritional value [19]. Saturated fats not only contribute to the development of metabolic diseases such as obesity and dyslipidemia, but have also been linked to the disruption of neurobiological mechanisms involved in mood regulation and eating behavior [19,20]. In adolescents, this developmental stage—characterized by hormonal, emotional, and social changes—can heighten vulnerability to these types of foods, particularly in situations of emotional stress or challenges in self-regulation [21].

Previous studies have shown associations between emotional eating and frequent consumption of foods high in saturated fat. For instance, a cross-sectional study conducted with women found that higher levels of emotional eating were positively associated with greater energy intake and more frequent consumption of items such as cakes, high-fat snacks, hydrogenated oils, and other calorie-dense foods [22]. Similarly, a study conducted with Taiwanese adolescents found that high levels of emotional eating were significantly associated with an increased likelihood of frequently consuming fast food, fatty snacks, processed meats, desserts, and sugar-sweetened beverages [23]. Furthermore, a study involving African American adolescents revealed that the relationship between emotional eating and dietary quality may be moderated by parental feeding practices, underscoring the critical role of the family environment in shaping emotional eating behaviors [24]. It should be noted that, although these studies provide valuable insights, most have focused on adult populations or culturally distinct settings, leaving a gap in our understanding of this relationship among Latin American adolescents. Therefore, it is essential to explore this link within local contexts, where emotional responses and eating behaviors may differ due to unique sociocultural influences.

Sleep is another factor increasingly associated with emotional eating [25]. Insufficient or poor-quality sleep can impair emotional regulation and heighten stress reactivity, thereby increasing the risk of maladaptive behaviors such as emotional eating [26]. Indeed, sleep disturbances have been associated with changes in the brain’s reward and appetite regulation systems, which may promote the intake of high-calorie foods [27].

There is growing evidence of the correlation between sleep quality and emotional eating, as well as its importance in young people and adolescents. For example, short sleep duration and increased rates of insomnia have been found to correlate with increased emotional eating in adolescents, specifically with anxiety, anger, and sadness [28]. On the other hand, in Latino adults living in the United States, López-Cepero et al. found that poor sleep quality is associated with increased emotional eating and that the correlation occurs mainly through negative emotions such as depression, anxiety, and perceived stress. Similarly, Zhou et al. [29] reported that, in Chinese university students, poor sleep quality is an indicator of increased emotional eating. The studies mentioned provide evidence that lack of sleep or poor sleep quality can alter emotional regulation and lead people to eat as an emotional response. Most of these studies have been conducted in adults or young Asians, highlighting the importance of studying this correlation in young Latin Americans, among whom sleep and sociocultural patterns can vary significantly.

During adolescence, BMI is particularly important, as this stage involves rapid changes in body composition that can influence body image, self-esteem, and eating behavior [30]. Furthermore, in the context of this study, it is worth mentioning that a high BMI could be linked to a higher risk of developing disordered eating patterns, such as emotional eating [31,32,33].

Some investigations have analyzed the association between emotional eating and elevated BMI, but the findings were contradictory, mainly in adolescents. A systematic review carried out by Limbers and Summers [31], which gathered 13 studies, led to the conclusion that, on the whole, there is no significant association between emotional eating and excessive weight in adolescents. Of the six longitudinal analyses examined, only one evidenced a significant prospective association between emotional eating and weight status. In fact, one intervention trial included in the review signaled that emotional eating at the beginning of the intervention was not significantly associated with weight modifications two years after bariatric procedures in obese adolescents. These findings suggest the hypothesis that, in contrast to what has been observed in adults, emotional eating would not be an immediate weight-gain determinant in adolescents. Yet, other studies introduce nuances [32]. Thus, Wilson et al. [33] observed that the relationship between perceived stress and emotional eating in student college populations was moderated by the BMI: emotional eating was more strongly associated with stress in those students with lower IMC, indicating that the effect of emotional stress on food consumption might vary according to whether or not another state of nutrition is considered. Taken together, these findings demonstrate that the association between emotional eating and BMI is intricate and may occur according to contextual elements such as stress level, access to coping resources, and the situation of development, which justifies continuing to explore this relationship in diverse adolescent populations.

Sociodemographic variables such as age and sex are also relevant. Age is a relevant factor in health, as it has an influence on the acquisition of healthy behaviors, including eating styles [34]. Thus, examining age as a variable associated with emotional eating enables us to understand how this behavior can be initiated or exacerbated during adolescent development.

Emotional eating changes as an individual ages, and emotions that trigger food consumption may change in the course of development, perhaps as a result of differences in emotional maturation [35]. In a study that compared children aged 9 to 10 years and young adults aged 19 years, it was observed that children had the desire to consume more snack foods when they were experiencing positive emotions, whereas young adults did so when they experienced negative emotions [36]. Likewise, Wu et al. [37] discovered that emotional eating is positively related to age among the female adolescents, while the relationship was not observed in the male adolescents. Also, in another study conducted with obese and overweight adolescents, it was found that greater instability in positive affect was linked with an increase in loss of control and overeating in the older individuals, whereas in the younger ones, this link was in the opposite direction [38]. These findings indicate that the impact of emotional instability on disordered eating behaviors may be higher as we progress in age.

While some studies point toward an increase in emotional eating with advancing age during adolescence, the empirical evidence is not conclusive, and the relationship between the two variables is not consistent. In one particular study, Heshmati et al. [39] found that age was not significantly correlated with emotional eating in a sample of American adolescents [39]. This result is consistent with earlier studies showing that age is not a valid predictor of emotional eating [40]. In fact, one study’s findings showed that age acts as a protective factor against emotional eating in adolescence, as in both negative and positive emotional contexts, older adolescents have less opportunity to use eating as a response to these emotions [41]. Consequently, there is the necessity to carry out additional research using multivariate models with the aim of clarifying the precise role played by age in the expression and persistence of emotional eating at this vital phase of development.

On the other hand, gender is a social determinant of health that influences how individuals perceive, deal with, and react to a variety of emotional and environmental situations [42]. The hormonal and biological differences between women and men, along with the social and cultural constructions of gender, can influence food habits and emotional coping mechanisms from early life [43,44]. Evidence indicates that adolescent females have a higher incidence of emotional eating compared to males, which has been linked to higher emotional reactivity, greater internalization of stress, and a more pronounced tendency to use food as an emotional regulation mechanism, especially in response to feelings of sadness or anxiety [36,37]. Additionally, it is hypothesized that women have more intense hedonic reactions to rewarding foods under negative emotional situations [36,37]. Gender disparities can be ascribed to both hormonal factors and sociocultural standards that lead to greater concern for body image and body appearance among women, which further increases their susceptibility to experience negative emotions and food use as an emotional compensatory factor.

Depression is one of the most common mood disorders during adolescence, a stage of life in which emotional vulnerability increases due to hormonal, social, and cognitive changes [45]. Depressive symptoms, such as depressed mood, loss of interest, fatigue, and feelings of worthlessness, not only compromise overall psychological functioning but can also have a negative effect on eating behavior patterns [46]. From this perspective, emotional eating has been thought of as an adaptive coping strategy in response to aversive affective states, such as depressive symptomatology, potentially leading to the establishment of unhealthy or disorganized eating patterns within this population [29].

Recent studies have strengthened the association between depressive symptoms and emotional eating among adolescents, despite differences in context and design. A systematic review and meta-analysis conducted by Muha et al. [47], which included 37 studies with 26,000 participants, confirmed the association between depressive symptoms and emotional eating, finding a moderate positive correlation as identified by cross-sectional studies (g = 0.48) and longitudinal studies (g = 0.37). In an investigation of young Mexican university students, Lazarevich et al. [48] found that emotional eating acted as an overall mediator of the relationship between depressive symptoms and body mass index (BMI), thereby substantiating the hypothesis that emotional distress can lead to maladaptive eating patterns that negatively influence nutritional status. Similarly, in recent multicenter studies, Silva et al. [49] discovered that depressive symptoms, anxiety, and stress were independently related to higher scores of emotional eating during the pandemic, thereby substantiating adolescents’ susceptibility to psycho-emotional risks during crisis conditions.

These findings consolidate the evidence on the relationship between depression and emotional eating, but also reveal the need for studies with culturally contextualized approaches to understand the underlying mechanisms in Latin American adolescent populations. In this context, it is necessary to examine this relationship considering other relevant variables, such as sleep, BMI, and consumption of high-fat foods, to generate a comprehensive view of the phenomenon.

Despite the growing interest in elucidating the factors that affect emotional eating in adolescence, the current evidence is fragmented and sometimes inconsistent [31]. Earlier investigations have sought to investigate the correlation between this behavior and other factors; however, a significant amount of this work has been conducted within specific cultural settings, essentially involving Western, adult, or clinical samples, thus limiting the generalizability of the findings. Furthermore, much of the literature has focused on the independent assessment of individual variables instead of integrating psychological, behavioral, and sociodemographic variables into a concerted predictive model.

In the context of Latin America, particularly in Peru, there is a pressing need for research that focuses on this issue among adolescent populations. This necessity arises from the high prevalence of overweight individuals, rapid changes in eating patterns, and mounting concerns regarding youth mental health. Moreover, there is a paucity of research addressing the potential moderating effects of variables such as gender or age on the relationship between individual factors and emotional eating. In light of the aforementioned limitations, the present study endeavors to make a meaningful contribution to the field by employing a predictive approach. This approach involves the examination of the joint association of six key variables: the consumption of foods high in saturated fat, sleep duration, BMI, symptoms of depression, age, and gender. The primary objective of this study is to elucidate the role of these variables in the phenomenon of emotional eating among Peruvian adolescents. The objective of this study is to identify the factors that exert the greatest influence on the predisposition to this behavior. The findings of this study will provide contextualized evidence that can serve as a basis for the development of future prevention and intervention strategies that are adapted to local realities. Therefore, the present study examines the association between saturated fat intake, sleep duration, BMI, symptoms of depression, age, and sex and emotional eating in a sample of Peruvian adolescents.

## 2. Materials and Methods

### 2.1. Design and Participants

The methodology employed in this study was a cross-sectional design, according to the classification proposed by Ato et al. [50]. With regard to the participants, a sample of individuals who participated in the study was used. This study encompassed a series of predictor variables, including the consumption of foods rich in saturated fats, sleep duration, BMI, and symptoms of depression. The dependent variable of the study was defined as emotional eating. The field study was conducted in three public educational institutions located in the district of Lima Este, Peru. The study sample consisted of 722 school-going adolescents who were selected through non-probabilistic intentional sampling based on criteria of accessibility and institutional willingness. The study subjects met the following inclusion criteria: they were between 12 and 18 years of age, they were enrolled in the selected educational centers, and they provided informed consent (and assent in the case of minors).

The minimum sample size was estimated using the Soper A-priori Sample Size Calculator for Multiple Regression (http://www.danielsoper.com/statcalc, accessed on 7 July 2025) [51]. The following parameters were established: a significance level of α = 0.05, a statistical power of (1 − β) = 0.95, an effect size f^2^ = 0.15, and six predictors (consumption of foods rich in saturated fats, sleep duration, BMI, symptoms of depression, sex, and age). The calculation yielded a minimum required sample size of 146 participants. The final study sample, consisting of 722 adolescents, exceeded this value, ensuring adequate statistical power for the analysis.

Table 1 presents the sociodemographic characteristics of the 722 adolescents. The mean age of the participants was 15.59 years (SD = 1.88). With respect to gender, the distribution was relatively balanced, with 51.2% of subjects identified as male and 48.8% as female.

### 2.2. Variables and Instruments

**Intake of foods rich in saturated fats:** Saturated fat intake was assessed using a food frequency questionnaire (FFQ). This FFQ was previously designed [52] and used in studies conducted in the Peruvian population [53]. The instrument consisted of 16 items that collected the frequency of consumption of foods commonly recognized as sources of saturated fats. The participants were instructed to indicate how frequently they performed a specified action, and the responses were recorded on an ordinal scale with the following categories: “Once a month or less” (0 points), “2 to 3 times a month” (1 point), “1 to 2 times a week” (2 points), “3 to 4 times a week” (3 points), and “5 or more times a week” (4 points). The total score, ranging from 0 to 64, was treated as a continuous variable in the statistical analyses to preserve variability and enhance explanatory power. The FFQ included items such as hamburgers, ground beef, fried chicken, bacon, sausages, cold cuts, hard cheeses, butter or margarine used in cooking, and pizza, which are recognized as the primary dietary sources of saturated fat. The questionnaire demonstrated high levels of reliability in its prior validation, with an ordinal alpha of 0.94, an omega coefficient of 0.94, and an H coefficient of 0.95, supporting its use for consistently measuring saturated fat consumption in Peruvian adolescents [54].

### 2.3. Duration of Sleep

Sleep duration was assessed by asking participants a direct question: “How many hours of sleep does your average day consist of?” Responses were classified into three categories, following criteria previously established in the scientific literature. Sleep was considered short when participants reported sleeping less than 7 h per day, normal when between 7 and 9 h, and prolonged when the duration of sleep was greater than 9 h [13,55].

**BMI:** Anthropometric assessment was carried out by a nutrition professional early in the morning over the course of a week, following standardized protocols. Participants were asked to remove their shoes and clothing as much as possible to ensure accurate data collection. The BMI was then calculated using the following formula: weight (kg) divided by height squared (m^2^). The adolescents were then classified according to the criteria established by the WHO, using BMI z-scores. According to this classification, underweight was defined as a BMI z-score less than –1, normal weight as a BMI z-score between –1 and +1, and overweight as a BMI z-score greater than +1 [56].

**Symptoms of depression:** Depressive symptoms were assessed using the Patient Health Questionnaire-2 (PHQ-2), which consists of the two core items from the PHQ-9 [57]: “Feeling down, depressed, or hopeless”, and “Having little interest or pleasure in doing things.” Participants were asked to indicate their experience over the past two weeks using a frequency scale ranging from 0 (no days) to 3 (almost every day). The total score ranges from 0 to 6, with a score of 3 or higher indicating clinically relevant depressive symptoms [58,59]. The scale has demonstrated acceptable internal consistency in the adult Peruvian population, with an alpha of 0.75 and an omega coefficient of 0.76 [60].

**Emotional Eating:** Emotional eating was assessed using the Spanish version of a specific questionnaire consisting of 10 items that probe the frequency with which participants eat in response to various emotions [61]. Each item was evaluated using a four-point Likert scale, with response options ranging from “never” (0) to “always” (3). The total score obtained allowed the adolescents to be classified into three levels of emotional eating: low or none (0–5 points), moderate (6–10 points), and high or very emotional (11–30 points) [62]. The instrument includes the following question: “Do you have particular dietary preferences or restrictions?” The questionnaire was originally developed in Spain and has been validated in the adolescent population in Chile [61,63]. Given that it has not yet been formally validated for Peruvian adolescents, the internal reliability of the scale was calculated in the total sample in the present study, obtaining a Cronbach’s alpha coefficient of 0.74, which is considered acceptable. According to the findings of previous studies conducted on adolescent populations, the levels of internal consistency reported were similar, with coefficients close to 0.71 [64].

### 2.4. Statistical Analysis

First, descriptive analyses were performed, reporting means, standard deviations, and absolute and relative frequencies. To examine differences in the study variables according to sex and age, Student’s *t*-tests for independent samples were applied, along with effect size calculation using Cohen’s d statistic and 95% confidence intervals. Normality was assessed using the Kolmogorov–Smirnov test and visual inspection of histograms and Q-Q plots. Homoscedasticity and linearity assumptions for the regression analysis were evaluated through residual plots. All assumptions were met. Subsequently, a multiple linear regression model was applied to identify significant predictors of emotional eating. The following were included as independent variables: consumption of foods rich in saturated fats, sleep duration (categories), z-BMI, symptoms of depression, age (categories), and sex. In addition, the assumptions of multicollinearity were verified using the TOL (Tolerance) and VIF (Variance Inflation Factor) indicators. Statistically significant values were determined as *p* < 0.05. The analysis was performed using IBM SPSS Statistics software version 26.

### 2.5. Ethical Considerations

The study was approved by the Research Ethics Committee of the Faculty of Health Sciences at the Universidad Peruana Unión (Ref. Code: 2025-CEB-FCS-UPeU-No.005. 7 January 2025), in accordance with the international ethical provisions established in the Declaration of Helsinki. Prior to data collection, formal authorization was requested from the participating educational institutions, as well as informed consent from the students’ parents or legal guardians. Informed consent was also obtained from the adolescents, who were informed about the objectives of the study, the voluntary nature of their participation, the confidentiality of the data, and their right to withdraw from the study at any time without consequences.

## 3. Results

As shown in Table 2, there were notable differences in some variables based on sex and age. The results of the study indicated that women reported having less sleep and higher levels of depression and emotional eating in comparison to men (*p* < 0.05), with moderate effect sizes. No differences were observed between sexes in saturated fat consumption or BMI. In terms of age, older adolescents (15–18 years) had higher BMI and emotional eating than those aged 12–14 years (*p* < 0.01). There were no significant differences in the other variables.

Table 3 presents the bivariate correlations among SFI, sleep duration, z-BMI, depressive symptoms, and emotional eating. Emotional eating was positively and significantly associated with saturated fat intake (r = 0.190, *p* < 0.001), depressive symptoms (r = 0.465, *p* < 0.001), and z-BMI (r = 0.114, *p* < 0.01), while it was negatively associated with sleep duration (r = –0.180, *p* < 0.001). Additionally, depressive symptoms showed significant positive correlations with SFI (r = 0.078, *p* < 0.05) and z-BMI (r = 0.024), and a negative correlation with sleep duration (r = –0.191, *p* < 0.001). Notably, SFI and z-BMI were not significantly correlated (r = –0.031), and sleep duration showed no meaningful association with BMI (r = 0.008).

As shown in Table 4, the findings of the multiple regression model for predicting emotional eating in adolescents are noteworthy. The model was found to be statistically significant overall, with an adjusted R^2^ value of 0.301 and a *p*-value less than 0.001. This indicates that the model explains 30.1% of the observed variance in emotional eating behaviors among adolescents. All predictors included in the analysis proved to be statistically significant. Higher saturated fat intake and higher levels of depressive symptoms were associated with higher emotional eating. Similarly, advanced age and female sex were shown to be associated with increased emotional eating tendencies. Conversely, sleeping seven hours or more exhibited a protective effect, significantly reducing this behavior. The model did not present multicolinearity problems (TOL > 0.69; VIF < 1.5), which supports its statistical validity.

## 4. Discussion

Adolescence is a critical stage in the formation of eating habits and the development of emotional regulation strategies. These aspects can be influenced by psychological, behavioral, and sociodemographic factors [1]. This study examined variables such as the consumption of foods rich in saturated fats, sleep duration, BMI, symptoms of depression, age, and sex to understand their relationship with emotional eating in Peruvian adolescents. The current study’s findings indicate a significant association between higher saturated fat consumption and higher levels of depressive symptoms with greater emotional eating. In a similar vein, being female and older were associated with a higher propensity for this type of behavior. Conversely, sleeping seven hours or more was associated with a reduced likelihood of emotional eating. The model demonstrated an overall explanatory power of 30.1% for the variance in emotional eating among adolescents.

One of the study’s primary findings was the identification of depression as the strongest predictor of emotional eating in adolescents. This result indicates that higher levels of depressive symptoms are associated with a greater tendency to use food as an emotional regulation strategy. These findings align with those reported in recent studies, which found a moderately strong positive association between depressive symptoms and emotional eating in adolescents, in both cross-sectional and longitudinal designs [47]. A similar study of university students demonstrated that symptoms of depression, anxiety, and stress are independently associated with an increase in emotional eating behaviors, underscoring the pivotal role of mental health in influencing eating patterns [49]. In the Peruvian context, a study we conducted among young adults yielded similar results [13]. However, while some studies have indicated a positive relationship, others have not reported significant associations [31]. These discrepancies can be attributed to methodological and contextual differences, as well as the influence of moderating factors such as social support, coping strategies, and the presence of other psychopathological symptoms.

Beyond the discrepancies, it has been suggested that depression can alter emotional regulation processes [65], increasing vulnerability to maladaptive eating behaviors [1,17]. Difficulty regulating emotions, a common characteristic in people with depressive symptoms, can encourage the use of food as an immediate strategy for emotional relief, as has been observed in contexts of induced hunger [66]. Furthermore, neurobiological alterations associated with depression, such as reward system dysfunction and hypothalamic-pituitary–adrenal axis dysregulation, have the potential to increase sensitivity to highly palatable foods [67]. This, in turn, could reinforce the vicious cycle between negative affect and overeating. These pathways underscore the necessity for interventions that integrate emotional management and the promotion of healthy eating habits as strategies to prevent eating disorders and mental health problems in adolescence.

Another salient finding was the positive association between the consumption of foods rich in saturated fats and emotional eating. This suggests that increased consumption of these foods is associated with a heightened tendency to overeat in response to negative emotions. These findings align with recent studies suggesting that highly palatable foods, particularly those high in saturated fat, function as hedonic reinforcers, providing temporary relief from unpleasant emotional states [22,68]. Furthermore, research conducted among adolescents indicates a correlation between emotional eating and increased consumption of ultra-processed foods [23], such as fatty snacks, pastries, and fast food items, which are high in saturated fat. However, the association between fat consumption and emotional eating may not be absolute, as food preferences can vary depending on cultural, economic, or food availability factors [69]. Therefore, while some cultures demonstrate a higher propensity for consuming fatty foods in response to emotions, others exhibit a stronger preference for sweet foods [70].

On the other hand, foods high in saturated fats have been shown to influence the brain’s reward system, leading to increased dopamine release and a sensation of well-being [71]. This rapid reward effect could serve as an immediate strategy for managing negative emotions [21]. Furthermore, repeated exposure to these types of foods in emotional contexts has the potential to reinforce behaviors that seek short-term gratification, making it challenging to establish eating patterns regulated by physiological signals of hunger and satiety [72]. This neurobehavioral mechanism could explain why adolescents who regularly turn to fatty foods in response to stress or sadness tend to maintain or intensify these patterns over time [21].

The results of the present study indicated that adolescents who slept seven hours or more exhibited a lower tendency for emotional eating compared to those who slept less than seven hours. This finding suggests that adequate sleep may offer a protective effect against emotional eating behaviors in this population. A recent study found that adolescents who slept less tended to experience emotional eating more often, a tendency that was particularly strong among those who were dissatisfied with their bodies [28]. A recent study of university students also found that poor sleep quality is a predictor of emotional eating, with depression playing a partial mediating role [29]. This lends further support to the notion that insufficient sleep has a negative impact on emotional regulation, which in turn, can lead to changes in eating patterns. However, the association between the two variables is not entirely consistent. In fact, certain cross-sectional studies have not identified a significant association between sleep duration and emotional eating [73,74]. These differences could be explained by the way sleep is measured (self-reported hours vs. objective assessments), the age range of the samples, or the presence of other uncontrolled psychological factors in the analysis.

In any case, from a physiological and behavioral standpoint, there are various mechanisms that could explain the observed relationships. For instance, sleep deprivation has been shown to alter appetite regulation systems, increasing levels of ghrelin (a hormone that stimulates hunger) and reducing levels of leptin (a hormone that promotes satiety), which increases appetite, particularly for high-calorie and high-fat foods [75]. Additionally, insufficient sleep has been shown to impair executive functions, leading to diminished self-control and emotional regulation. This can result in impulsive reactions, such as overeating in response to stress or negative emotions [76]. Therefore, it is important to promote proper sleep hygiene as a strategy to prevent maladaptive eating behaviors in adolescents.

Regarding BMI, the results of this study indicated a positive association between higher BMI and higher levels of emotional eating in adolescents. Although the strength of this relationship was lower compared to other variables, the finding suggests that overweight adolescents may be more vulnerable to using eating as a strategy to cope with negative emotional states. This result is consistent with studies such as that of Lazarevich et al. [48], who reported that emotional eating mediated the relationship between depressive symptoms and higher BMI in young Mexican adults. Similarly, a systematic review concluded that, although longitudinal evidence is limited, several cross-sectional studies have found associations between emotional eating and higher body weight in adolescents, although not universally [31]. Indeed, research indicates that the association between emotional eating and weight gain can be influenced by external factors, including the level of social support or coping mechanisms available [33]. In some contexts, it has been observed that high BMI does not necessarily predict higher emotional eating independently. Rather, its impact may be modulated by psychological variables such as perfectionism [77].

Among the possible underlying mechanisms, it has been suggested that adolescents with higher BMI may experience greater social pressure and body stigmatization, which generates negative emotions that, in turn, encourage emotional eating as a coping mechanism [77]. In addition, being overweight may be associated with more dysfunctional dietary patterns—such as higher consumption of ultra-processed and high-fat foods—that perpetuate the cycle between emotional distress and overeating [32]. On the other hand, alterations in the brain’s reward systems could also favor a more intense response to high-calorie foods in adolescents with greater adiposity, reinforcing the relationship between BMI and emotional eating [27,71].

Finally, the present study found that both gender and age were significant predictors of emotional eating. In particular, being female and older were associated with higher levels of this behavior. These findings are consistent with previous research that has documented a higher prevalence of emotional eating in women compared to men [36,37]. In fact, it has been suggested that adolescent girls tend to report greater use of maladaptive emotional strategies, including eating [8,78], possibly due to greater emotional sensitivity and higher levels of perceived stress during this stage of development.

Regarding age, the findings suggest that, as adolescents mature, they are more prone to using food as a means of emotional regulation. This pattern has also been reported by Wu et al. [37], who found that the relationship between age and emotional eating was positive in adolescent females but not in males. This suggests that emotional development and hormonal changes during puberty may intensify vulnerability in girls as they become older. Also, Moss et al. [36] observed discrepancies in the emotions that precipitated emotional eating in children and young adults, underscoring that the impact of age might also be contingent on the prevailing emotion. These findings suggest that, as adolescents transition into adulthood, heightened academic, social, and familial pressures may amplify their exposure to stressors, prompting them to utilize food as a coping mechanism for managing adverse emotions, despite the absence of more effective regulatory skills. However, not all studies concur entirely with this trend. In some cultural contexts or among specific populations, weak or non-significant associations between age, gender, and emotional eating have been reported [32,39,40]. These findings could reflect variations in sociocultural norms related to emotional expression, access to food, and learned coping models.

### 4.1. Limitations and Future Considerations

The findings of the current study should be interpreted considering several limitations. First, because we conducted a cross-sectional study, we cannot claim that the associations observed between the explanatory variables (sleep duration, BMI, depression, age, and sex) and the outcome variable (emotional eating) are causal. Therefore, longitudinal studies are suggested to better understand the direction and evolution of these relationships. Second, all variables were assessed using self-report, which can introduce social desirability bias or recall errors, particularly in measures such as sleep duration or food consumption. Furthermore, in the specific case of depression, we did not use clinical diagnoses of psychological symptoms made by a physician based on medical care data. This is especially important because the gold standard for establishing a psychiatric diagnosis requires a structured interview and functional neuroimaging [79,80,81]. Thirdly, although a questionnaire validated in other Spanish-speaking populations was used to measure emotional eating [61,63], we do not yet have a specific validation for Peruvian adolescents; therefore, this aspect must be taken into account when interpreting some items from a cultural perspective. Finally, the sample was limited to three public schools in eastern Lima; private schools and other educational institutions located in other geographical regions of Peru were not considered. Consequently, the results should be generalized to other geographical or socioeconomic contexts with caution. Future studies should broaden the geographic, cultural, and socioeconomic diversity of the samples, and incorporate clinical assessments of mental health along with objective measurements of variables such as sleep and dietary intake. This approach will help strengthen the empirical foundation and support the development of more specific and culturally relevant interventions to prevent emotional eating and promote comprehensive health among adolescents.

### 4.2. Implications for Public Health

Despite these limitations, we believe that this study provides valuable information that can serve as a basis for future research and interventions carried out in both school and community settings, aimed at promoting the mental and nutritional health of adolescents. Specifically, it is worth mentioning that the results of the current study support the design of strategies to prevent unhealthy eating behaviors and promote health among adolescents. In fact, since saturated fat consumption, inadequate sleep duration, high BMI, depressive symptoms, female gender, and older age are factors associated with emotional eating, policies and interventions should consider a multifactorial approach that integrates not only nutritional aspects but also emotional and behavioral aspects. Furthermore, since adolescents constitute a significant school population, it is essential that educational institutions promote not only healthy eating habits but also adaptive emotional regulation strategies, educating students about the importance of adequate sleep and encouraging psychosocial support activities aimed at adolescents. In addition, promoting the importance of mindful eating and depression prevention is suggested, as these could contribute to reducing the incidence of emotional eating and, consequently, reduce the risk of excess body weight and long-term metabolic disorders.

## 5. Conclusions

This study provides evidence on the factors associated with emotional eating in Peruvian adolescents, highlighting the fundamental role of depressive symptoms, consumption of foods rich in saturated fats, sleep duration, BMI, sex, and age. The findings underscore the multifactorial nature of emotional eating and the need to address both psychological and behavioral determinants in health promotion strategies targeting this population.

## Figures and Tables

**Table 1:** Description of the sociodemographic characteristics of the study sample (*n* = 722).

Characteristic	*n*	%
**Age**	M = 15.59	SD = 1.88
**Sex**		
Female	352	48.8
Male	370	51.2

Note. M = mean; SD = standard deviation.

**Table 2:** Analysis of variables related to consumption of foods rich in saturated fats, sleep, BMI, depression, and emotional eating according to sociodemographic variables.

Variables	SFI	Sleep	z-BMI	Depression	Emotional Eating
**General M (SD)**	14.10 (6.94)	1.99 (0.79)	23.91 (3.90)	2.48 (1.77)	9.42 (5.41)
**Sex ^1^**					
*p*	0.095	0.028 *	0.379	<0.001 ***	<0.001 ***
95% CI	[−1.873, 0.153]	[−0.242, −0.014]	[−0.315, 0.826]	[0.791, 1.286]	[2.611, 4.116]
Cohen’s d	−0.124	−0.164	0.066	0.613	0.654
**Age ^1^**					
*p*	0.885	0.796	<0.001 ***	0.930	0.007 **
95% CI	[−1.092, 0.942]	[−0.130, 0.099]	[−2.412, −1.301]	[−0.248, 0.271]	[−1.877, −0.299]
Cohen’s d	−0.011	−0.019	−0.489	0.007	−0.202

Note. M = mean; SD = standard deviation; Student’s *t*-test ^1^; z-BMI, body mass index z-score; CI = confidence interval at 95%; *p* = *p*-value; SFI = saturated fat intake, * *p* < 0.05, ** *p* < 0.01, *** *p* < 0.001.

**Table 3:** Correlation analysis between consumption of foods rich in saturated fat, sleep, z-BMI, depression, and emotional eating.

Variables	1. SFI	2. Sleep Duration	3. z-BMI	4. Depression	5. Emotional Eating
1. SFI		0.068	−0.031	0.078 *	0.190 ***
2. Sleep Duration	-	-	0.008	−0.191 ***	−0.180 ***
3. z-BMI	-	-	-	0.024	0.114 **
4. Depression	-	-	-	-	0.465 ***
5. Emotional Eating	-	-	-	-	-

Note. SFI = saturated fat intake, z-BMI = body mass index z-score, * *p* < 0.05, ** *p* < 0.01, *** *p* < 0.001.

**Table 4:** Factors associated with emotional eating in Peruvian adolescents.

Model		Standardized Coefficients		t	TOL	VIF	*p*
		β	CI				
1	(Constant)			−1.24			0.214
	SFI	0.186	[0.124, 0.248]	5.90	0.978	1.02	<0.001 ***
	Sleep (<7 h)	Reference					
	Sleep (7 h)	−0.094	[−0.167, −0.020]	−2.50	0.691	1.45	0.013 *
	Sleep (>7 h)	−0.126	[−0.199, −0.052]	−3.37	0.697	1.43	<0.001 ***
	z-BMI	0.082	[0.019, 0.146]	2.53	0.922	1.08	0.011 *
	Depression	0.365	[0.299, 0.431]	10.84	0.855	1.17	<0.001 ***
	15 to 18 years	0.083	[0.019, 0.146]	6.33	0.903	1.11	<0.001 ***
	Being female	0.208	[0.143, 0.272]	2.55	0.921	1.09	0.011 *

Note. Dependent variable: emotional eating; SFI = saturated fat intake; z-BMI = body mass index z-score; Model 1: Adjusted R^2^ = 0.301, ANOVA F (F = 45.276, *p* < 0.001). * *p* < 0.05, *** *p* < 0.001.

## Data Availability

The data that support the findings of this study are available on request from the corresponding author. The data are not publicly available due to privacy or ethical restrictions.

## Abbreviations

The following abbreviations are used in this manuscript:

SFI	Saturated fat intake
z-BMI	Body mass index z-score
SD	Sleep duration
EE	Emotional eating
